# Current understanding of the interplay between extracellular matrix remodelling and gut permeability in health and disease

**DOI:** 10.1038/s41420-024-02015-1

**Published:** 2024-05-27

**Authors:** Aurora Vilardi, Stefan Przyborski, Claire Mobbs, Alessandro Rufini, Cristina Tufarelli

**Affiliations:** 1 Cancer Research Centre, University of Leicester, Leicester, LE2 7LX United Kingdom; 2 Department of Biosciences, Durham University, Durham, DH1 3LE United Kingdom; 3 Department of Biosciences, University of Milan, Milan, 20133 Italy

**Keywords:** Inflammatory bowel disease, Mechanisms of disease, Cell adhesion

## Abstract

The intestinal wall represents an interactive network regulated by the intestinal epithelium, extracellular matrix (ECM) and mesenchymal compartment. Under healthy physiological conditions, the epithelium undergoes constant renewal and forms an integral and selective barrier. Following damage, the healthy epithelium is restored via a series of signalling pathways that result in remodelling of the scaffolding tissue through finely-regulated proteolysis of the ECM by proteases such as matrix metalloproteinases (MMPs). However, chronic inflammation of the gastrointestinal tract, as occurs in Inflammatory Bowel Disease (IBD), is associated with prolonged disruption of the epithelial barrier and persistent damage to the intestinal mucosa. Increased barrier permeability exhibits distinctive signatures of inflammatory, immunological and ECM components, accompanied by increased ECM proteolytic activity. This narrative review aims to bring together the current knowledge of the interplay between gut barrier, immune and ECM features in health and disease, discussing the role of barrier permeability as a discriminant between homoeostasis and IBD.

## Facts

- Increased barrier permeability represents a feature of inflammatory diseases affecting the intestine, such as IBD.
- Chronic unresolved inflammatory events relate to increased ECM remodelling, mainly due to matrix metalloproteinases (MMPs).
- MMPs-2, -7, -9, -12 and -13 favour pro-inflammatory signalling pathways and increased barrier permeability.
- Activation of T helper cells 1 (Th1), Th2, Th17 and Th9 has been observed concomitantly to increased barrier permeability.

## Open questions

- Can a detailed knowledge of the anti-inflammatory immune cells, cytokines and their signalling pathways be exploited to develop treatments for IBD?
- Can a better understanding of the ratio between MMPs and TIMPs in different conditions improve the development of new clinical applications?
- Can beneficial microbial phyla reverse ECM remodelling and/or dampen the proteolytic activity in IBD?
- Can intestinal in vitro 3D models be used in IBD research to overcome the physiological and ethical limitations of animal models?

## Introduction

**Fig. 1:**
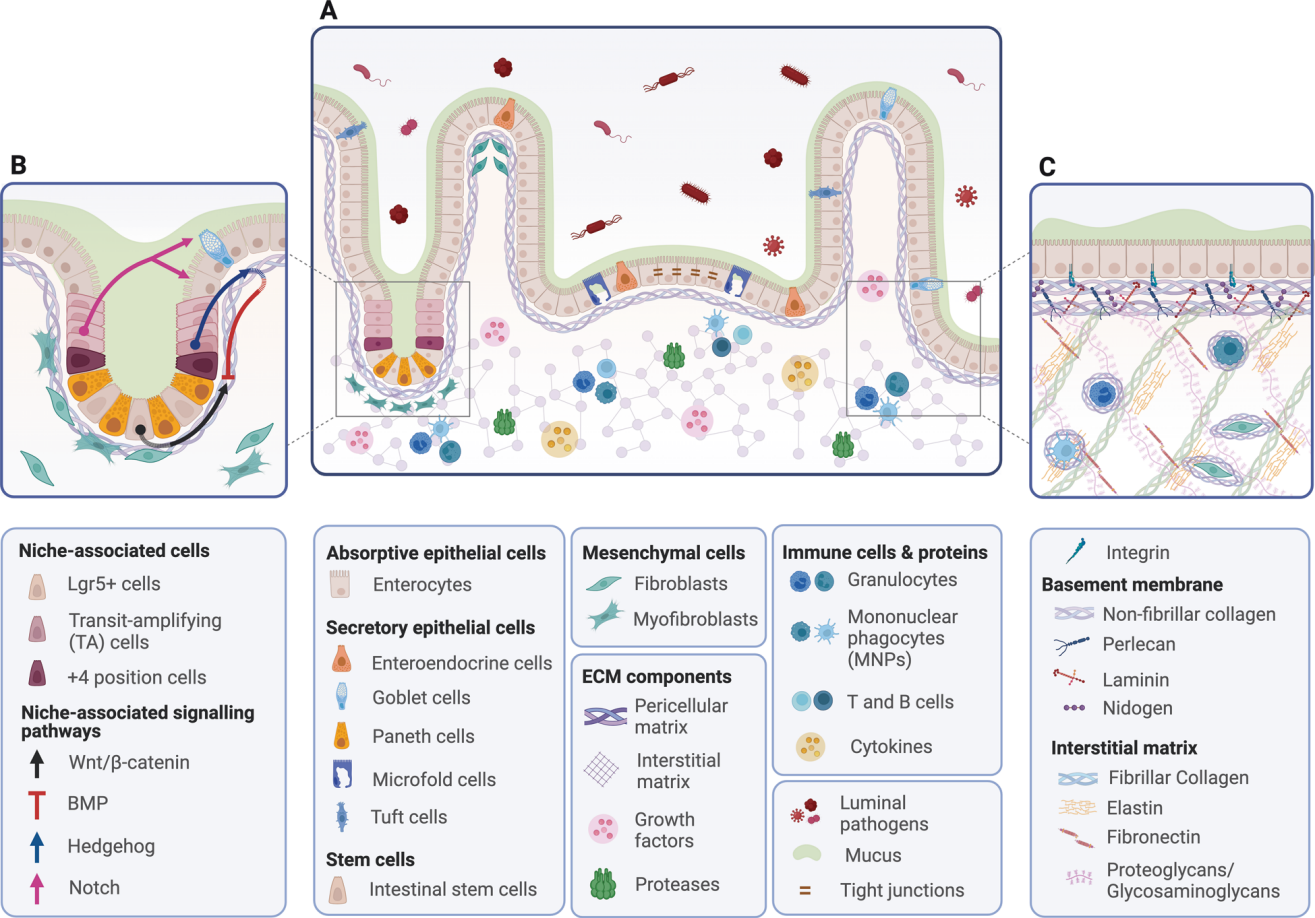
Schematic of the intestinal wall architecture under physiological conditions (not to scale). **A** The epithelial barrier represents the first line of mechanical separation between the lumen and the intestinal mucosa. A polarised layer of epithelial cells lies on the complex mix of molecules of the ECM, which gives biophysical support and contributes to molecular signalling. The epithelial and ECM compartments maintain a fine balance by interacting with the mesenchyme, which contributes to tissue remodelling and repair, and adaptation to bacterial stimuli. **B** The interplay between intestinal stem cells (ISCs) and the mesenchymal compartment defines the environment of the crypt niche. Paneth cells and mesenchymal cells (MCs) maintain a fine balance between promoting Wnt/β-catenin and inhibiting BMP pathways, allowing constant epithelial renewal. Once ISCs differentiate into transit-amplifying (TA) cells, asymmetric activation of the Notch signalling pathways is required for cell differentiation into absorptive or secretory lineages. When newly differentiated cells are generated, Hedgehog signalling is activated, promoting BMP pathways, thus stopping further differentiation of epithelial cells [36]. **C** The ECM is a scaffolding structure of the intestinal wall characterised by a pericellular matrix (PM) and an interstitial matrix (IM). PM is composed of fibrous proteins linked by crosslinking enzymes, such as lysyl oxidases (LOX), whose role is to surround and support cells. A type of pericellular matrix specific to epithelial and endothelial cells is the basement membrane, mainly characterised by laminins, collagen IV, perlecan and nidogen. IM is made of fibrous proteins rich in glycosaminoglycan elements and non-fibrous proteins, whose distribution within cells allows them to crosstalk and interact [21]. Created with BioRender.com.

**Fig. 2:**
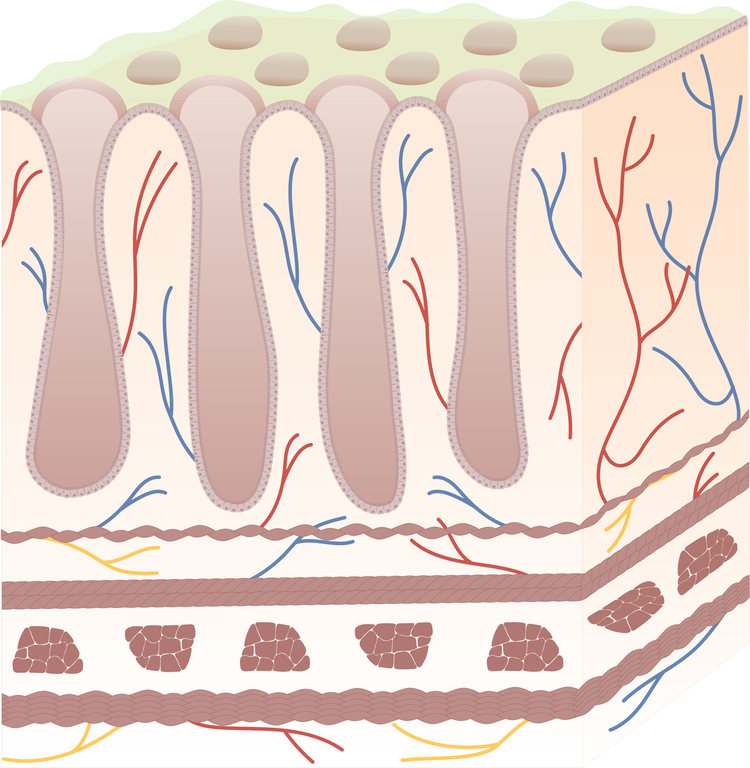
Structural features of the intestinal wall layers. The intestine has a highly specialised surface known as the intestinal wall, whose dual function is to avoid tissue infiltration and colonisation by pathogens, while allowing the absorption of nutrients (small intestine), water (large intestine) and ions (both small and large intestine). The intestinal wall is a complex structure comprising four tissue layers: the mucosa directly in contact with the lumen, followed by submucosa, muscularis propria and serosa [133]. This compartmentalisation reflects the different distribution of connective, neural and vascular components. Whilst serosa and muscularis propria are mainly characterised by neural fibres, connective tissue and smooth muscle cells; submucosa and mucosa host, but are not limited to, lymphatic vessels, connective tissue and epithelial cells [134]. Created with Adobe Illustrator.

The intestinal wall is a complex structure that ensures the integrity and functionality of the intestinal epithelium (Fig. 1). It does so by exerting a dual function: avoiding tissue infiltration and colonisation by pathogens while enabling intestinal permeability, i.e. the regulated passage of water, nutrients, and ions across the epithelial barrier [1]. Intestinal permeability is modulated by tight interactions among epithelial cells, crypt-associated signalling pathways monitored by mesenchymal cells (MCs), and extensive crosstalk between epithelial cells and components of the extracellular matrix (ECM) [2, 3]. Under physiological conditions, occasional damage to the epithelium triggers a series of restorative signalling pathways. In this context, the tissue mesenchyme orchestrates finely-regulated proteolysis of the ECM by proteases, such as matrix metalloproteinases (MMPs), which play a major role in remodelling the scaffolding tissue and epithelial restoration [4]. Intestinal inflammatory conditions result in dysregulated crosstalk between epithelial cells and ECM, which is associated with increased proteolytic activity, as well as higher intestinal permeability [5]. Inflammatory bowel disease (IBD) is a group of non-infectious, chronic, and relapsing-remitting inflammatory conditions of the gastrointestinal tract, including Crohn’s disease (CD) and Ulcerative Colitis (UC). Although their exact aetiology remains unknown, genetic, environmental, microbial, and immune factors are known to play a role in disease development [6]. CD and UC share similar symptoms, such as abdominal pain, fever, vomiting, diarrhoea, rectal bleeding, weight loss and anaemia [7]. However, the affected tissue area and treatment regime differ between the two diseases. For example, CD is characterised by transmural and discontinuous inflammation across the whole intestine, whereas UC involves mucosal and submucosal inflammation mainly restricted to the colon [8] (Fig. 2). Table 1 outlines the main differences between UC and CD in terms of histological and inflammatory signatures, focusing on the role of ECM and MMPs.

**Table 1:** Pathological, inflammatory, and cellular and immunological differences between ulcerative colitis (UC) and Crohn’s disease (CD).

	Crohn’s disease (CD)	Ulcerative colitis (UC)	Ref
Gastrointestinal (GI) symptoms	Abdominal pain, fever, vomiting, diarrhoea, rectal bleeding, weight loss, anaemia		[7]
Location	Any region of the GI	Terminal region of the colon	[127]
Disease distribution	Diffuse	Continuous	[127]
Epithelial architecture	Preserved	Crypt fission and distortion	[10, 128]
Type of inflammation	Transmural with abscesses, strictures, fistulae and granulomas	Mucosal and submucosal	[8]
Adaptive immune phenotype	Th1, Th17 (IL-17, IL-23, IL-32)	Th2 (IL-5, IL-13, IL-15, IL-33)	[52, 70, 129]
ECM proteins	Higher serum levels of laminin^a^, collagen fragments (C3M, C4M), sulphated glycosaminoglycans^b^, elastin fragments (NE-EL) and biglycan (Pro-C5, C5M)	Higher serum levels of fibronectin^a^, hyaluronan and collagen fragment (C1M)	[13, 86, 129]
	Collagen fragments (Pro-C5M, C5M)		
MMPs expression	MMP-1, -2, -3, -7, -8, -9, -10, -12, -13, -14		[77, 130]

^a^Both before and after 1 year of treatment with monoclonal antibodies.

^b^After 1 year of treatment with steroidal anti-inflammatory drugs.

In the present narrative review, we aim to summarise the current knowledge on the compartmentalisation and function of the intestinal wall, focusing the discussion on features of barrier permeability related to the immune network and the ECM environment, with a particular emphasis on MMPs.

## The intestinal wall: compartmentalisation and functions

### The intestinal epithelium

**Fig. 3:**
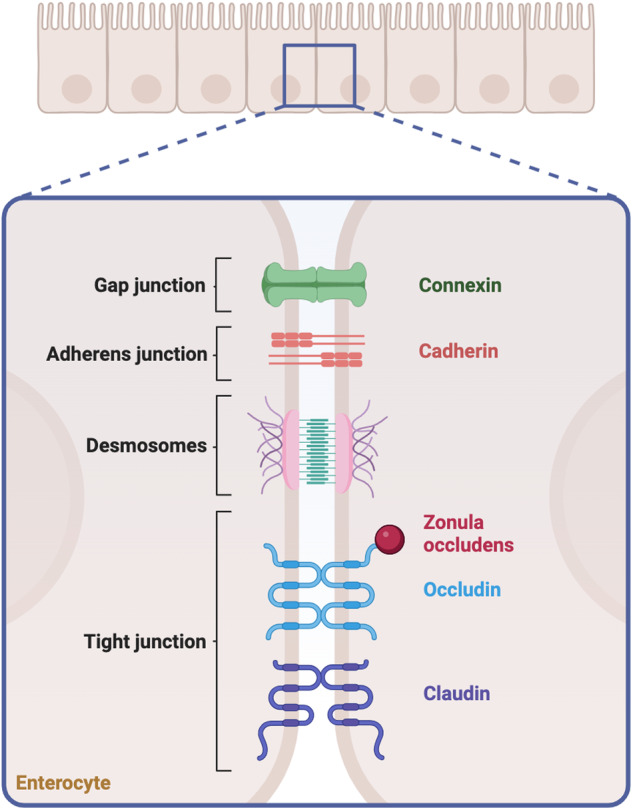
Junctional network involved in intestinal paracellular transport. TJs are classified into transmembrane and cytoplasmatic proteins. Transmembrane proteins include TAMP (TJ-associated MARVEL proteins), such as occludins, tricellulin and marvelD3, and claudins, that monitor the movement across the barrier establishing its semipermeable properties; Junctional adhesion molecules (JAMs), that sustain the TJs actively involved in paracellular pathways; and angulins, that act as regulators. Cytoplasmatic proteins include the zonula occludens (ZO) family protein, which anchors transmembrane proteins to cytoskeletal components [46]. A similar role is dictated by desmosomes and adherens junctions—belonging to the Cadherins family—that provide interaction sites and mechanical strength. In addition, gap junctions allow cell communication by releasing proteins, such as Connexin. A comprehensive description of TJs and their relation to inflammatory signalling pathways has been previously reviewed by [135]. Created with BioRender.com.

The intestinal epithelium is shaped into villi, epithelial projections that increase the intestinal surface area, and epithelial invaginations known as crypts of Lieberkühn, that act as gatekeepers for epithelial regeneration and homoeostasis by harbouring intestinal stem cells (ISCs) [9, 10]. This morphological architecture determines the absorptive and secretory functions of the intestinal epithelium, whereas intestinal barrier selectivity is controlled by transcellular and paracellular movements across the epithelial layer. Transcellular movements are determined by size- and charge-selective channels and transporters; paracellular movements exploit the physical spaces between adjacent enterocytes and are regulated by intercellular junctions, including tight junctions (TJs), adherens junctions and desmosomes [11, 12] (Fig. 3). Intestinal epithelial cells (IECs) are arranged to form a biological barrier and are the first line of defence of the intestinal wall. Most of the intestinal epithelium is made of absorptive enterocytes within the villi, interspersed with enteroendocrine cells, which are responsible for releasing hormones; goblet cells, which secrete a protective hydrogel layer, the mucus, and its related proteins, mucins; and tuft cells, involved in adaptive immunity. Other types of epithelial cells localised within the intestinal crypts are the Lgr5+ ISCs, which ensure epithelial repair and self-renewal; Paneth cells, interspersed among ISCs, which contribute to ISCs turnover and secrete antimicrobial peptides; +4 position cells, relatively quiescent stem cells with protective roles towards Lgr5+ ISCs damage; and transit-amplifying (TA) cells that inhabit the upper half of the crypt and are progenitor cell types committed to differentiating into specialised cells of the villi [12–16] (Fig. 1A).

### The extracellular matrix

The ECM is a dynamic network of proteins, growth factors and degrading enzymes that play a pivotal role in supporting and protecting the tissue integrity and epithelial layer. ECM components are mainly secreted by the mesenchymal cells with contributions from epithelial, endothelial and immune cells [17, 18]. ECM proteins can be classified as fibrous and non-fibrous. Fibrous proteins include type I-X and XIV collagens; and glycoproteins, such as laminins, elastins, fibronectin, nidogens and tenascin. Non-fibrous proteins comprise proteoglycans, such as heparan sulfate proteoglycans (HSPGs) (e.g. perlecan, syndecans); keratan sulfate; chondroitin/dermatan sulfate (e.g. decorin, biglycan); and glycosaminoglycans, such as hyaluronan [19, 20]. While fibrous proteins work as solid pillars to support the intestinal architecture, non-fibrous proteins allow cell–cell interactions, facilitated by the interplay between their core proteins and cellular surface receptors, such as integrins and growth factor receptors [21]. Specifically, collagens contribute to epithelial tensile strength and elasticity; glycoproteins and proteoglycans are responsible for epithelial-ECM and epithelial-mesenchymal crosstalk, cell proliferation, adhesion, migration, differentiation and survival; and glycosaminoglycans maintain ECM assembly and hydration [5] (Fig. 1C).

### ECM turnover

The continuous process of ECM turnover is crucial for maintaining tissue homoeostasis and regulating mechanical changes, such as shear and stretch, along the intestinal wall [22]. The turnover of ECM proteins is enzymatically regulated by ECM proteases, degrading enzymes belonging to the metzincin family, including matrix metalloproteinases (MMPs), $\alpha$-disintegrin and metalloproteinases (ADAMs) and $\alpha$-disintegrin and metalloproteinases with thrombospondin motifs (ADAMTSs) [23]. Among them, the most relevant are MMPs, a family of 23 zinc-dependent endopeptidases consisting of a propeptide, a catalytic metalloproteinase region, and a hinge and hemopexin domain [24]. MMPs include collagenases (MMP-1, -8, -13, -18), gelatinases (MMP-2, -9), stromelysins (MMP-3, -10, -11), matrilysins (MMP-7, -26), membrane-type enzymes (MT1-6-MMP), and macrophage elastase (MMP-12) [25, 26].

### Regulation of the activity of MMPs

Activation of MMPs is regulated by (i) the processing of their inactive precursors, known as pro-MMPs; (ii) their specific location; and (iii) their inhibition by endogenous or exogenous MMPs inhibitors. Pro-MMPs retain a cysteine in the propeptide domain linked to an atom of Zn^+^ in the catalytic domain. The cysteine-Zn^+^ complex has been established as a latency mechanism that maintains the enzymes in an inactive state. However, cross-activation within MMPs and other proteases can remove the binding between the cysteine and the Zn^+^, resulting in a “cysteine switch” and subsequent MMP activation [27, 28]. Once activated, the activity of MMPs is largely controlled by tissue inhibitors of metalloproteinases (TIMPs). Mammalian TIMPs are classified into TIMP-1 to -4 [23, 29, 30]. While TIMP-2 is ubiquitously expressed throughout the body, TIMP-1, -3, and -4 expression is inducible in specific tissues [31]. Overall, TIMPs can bind the majority of the MMPs with a limited selectivity [32]. TIMP-1 preferentially regulates MMP-1, -2, and -9, while TIMP-2 controls MMP-2 and some members of the MT-MMPs [24].

### Roles of MMPs in ECM remodelling

The regular interaction between ECM proteins, proteases, and protease inhibitors contributes to defining the protease:antiprotease ratio, which determines the rate of ECM remodelling [23, 29, 30].

The role of MMPs in tissue homoeostasis is exemplified by mouse embryonic fibroblasts from *Mmp2* null mice. Forced expression of the human MMP-2 gene in these cells was able to activate the transforming growth factor beta (TGF*-β*) and the connective tissue growth factors (CTGF) by releasing them from their latency complexes [25]. Indeed, CTGF remains in an inactive state by forming a complex with vascular endothelial growth factor (VEGF). However, cleavage of this inhibitory complex by MMP-2 results in the release of CTGF and ECM deposition [33]. Moreover, studies on human cell lines highlighted how ECM remodelling driven by MMPs also influences the fate of MCs, allowing differentiation into adipogenic, chondrogenic, osteogenic, and endothelial lineages. This is supported by several studies that highlighted how increased expression of ECM fibres, often remodelled by MMPs (e.g. MMP-2, -9, and -13), allowed active differentiation (reviewed in [26]).

### The mesenchymal compartment

Additional monitoring of the functionality of epithelial cells and ECM is provided by MCs, an umbrella term including smooth muscle cells, pericytes, interstitial cells of Cajal and submucosal fibroblasts, which regulate gut motility, vascular and lymphatic support, and lymphangiogenesis [34]. Fibroblasts and myofibroblasts are integral to intestinal structure and function and are involved in controlling intestinal morphology and architecture, tissue compartmentalisation, cell interactions, wound healing, and immune cell turnover [35]. To allow epithelial renewal and ISCs turnover, MCs, as well as epithelial cells, produce Wnt, Notch and Hedgehog ligands, epidermal growth factor (EGF), inhibitors of the bone morphogenic pathways (BMP) and prostaglandin E2 (PGE2) [36, 37]. By contributing to balancing these signalling pathways, MCs allow the differentiation of ISCs into transit-amplifying (TA) cells first and absorptive and secretory epithelial lineages later. This ensures epithelial renewal every 3–5 days under physiological conditions and favours tissue repair following injury [36–38] (Fig. 1B). Single-cell RNA sequencing (scRNA-seq) of human colonic biopsies identified distinct clusters of fibroblasts involved in crypt architecture by expressing genes essential for stem cell functionality [39, 40]. Additional scRNA-seq studies confirmed the regenerative features of MCs in healthy tissues and observed their potential to promote inflammatory markers release, immune migration and response to bacterial stimuli in newly diagnosed UC patients [39].

## Investigating barrier permeability: from balance to IBD

The integrity of the intestinal epithelium represents a pivotal factor that discriminates between homoeostatic and pro-inflammatory conditions. Barrier permeability and IBD are tightly associated; however, whether the leakiness of the barrier is the cause or consequence of the wider mucosal damage is not yet completely understood. For example, asymptomatic IBD patients, as well as their healthy first-degree relatives, exhibit increased gut permeability—followed by the later onset of CD for the second group —suggesting that early barrier leakiness might be a trigger for disease development [41, 42].

While a limited number of brush transporters, expressed on the apical membrane of intestinal epithelial cells, facilitate transcellular movement through the epithelium, the primary factor influencing barrier permeability is the paracellular movement between adjacent cells [43, 44]. The paracellular transport is governed by the apical junction complexes previously shown in Fig. 3. Under physiological conditions, these complexes permit the passage of molecules through the ‘pore’ and the ‘leak’ pathways, which differ in the capacity and the size of the crossing molecules. The pore pathway has a high capacity for low-molecular-weight molecules, while the leak pathway allows the passage of high-molecular-weight molecules at a lower capacity [45]. Distinct mechanisms govern the two pathways. In the pore pathway, claudins regulate the passage of molecules; in the leak pathway, the movement is also governed by cytoskeletal forces, in addition to the interactions between transmembrane proteins (e.g., claudins, occludins and JAMs). Although there is some controversy about the leak pathway, with several studies suggesting that it is a mere consequence of transient injury to the epithelium, its existence is somehow supported by the fact that no cellular death or evident damage has been thus far associated with certainty to the pathway (reviewed in [46]). Nonetheless, in case of persistent damage to the epithelium, the regulation of the barrier permeability is compromised, and a continuous flux of molecules moves across the barrier, exposing the intestinal mucosa to a higher amount of pro-inflammatory antigenic stimuli. This type of uncontrolled transport, known as the ’unrestricted pathway’, contributes to establishing barrier leakiness as a pathophysiological hallmark of intestinal diseases, such as IBD [47].

### The gut immune microenvironment: from homoeostasis to IBD

The epithelial barrier functions as a bridge between luminal antigens and the inner gut-associated lymphoid tissue (GALT), the largest lymphoid organ in the body [48]. Several pathways enable the intestinal epithelium to present luminal antigens to the immune system, such as enterocyte-dependant transport of small molecules, vesicle-mediated uptake by goblet cells, dendritic internalisation by macrophages and enteroendocrine recognition [43]. An important role is exerted by microfold (M) cells interspersed among the IECs. M cells serve as priming centres for immune responses by promoting antigen sampling to the underlying immune environment through dendritic cells and macrophages [49]. Following interaction with neighbouring Peyer’s patches (PP) and isolated lymphoid follicles, antigens are screened by mesenteric lymph nodes (MLN), the final checkpoint that discriminates between suppressive or stimulatory immune responses [48].

**Fig. 4:**
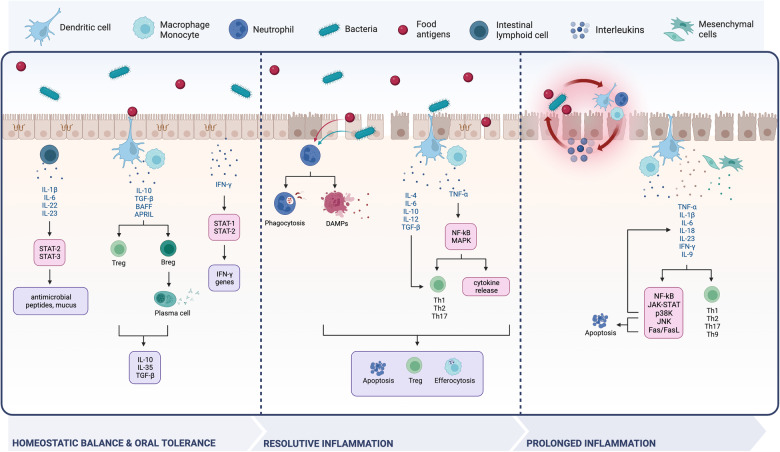
The immune environment of the intestinal wall from balance to disease. The structural status of the intestinal barrier affects the tendency of immune cells to release cytokines (represented in blue) that stimulate transcription factors (pink boxes) and signalling pathways, causing the activation of inflammatory stimuli and cellular components (purple boxes). These are not just a mere consequence but could also represent a trigger of the inflammatory cascade hereby represented, which depicts the difficulty of untangling this process. When the intestinal barrier is healthy and selectively permeable, tolerant signalling takes place. Transient or persistent gaps in the barrier promote either resolutive or detrimental inflammation. Created with BioRender.com.

A determinant of the GALT’s tolerogenic *vs* inflammatory response is represented by the nature and the amount of crossing antigens, which depend on the mechanisms of epithelial transport and the status of the barrier. When pore and leak pathways function regularly, the mucosal immune response is shifted towards homoeostatic balance and suppressive functions. If transient damage to the epithelium occurs, inflammation is triggered and resolved; however, if the damage persists, the unrestricted pathway takes place and initiates chronic inflammation (Fig. 4).

In health (Fig. 4, left panel), a significant role is played by the STAT family of transcription factors, usually activated in epithelial and immune cells through phosphorylation by Janus protein tyrosine kinase (JAK) following cytokine stimulation [47]. In innate lymphoid cells (ILCs), interferon-gamma (IFN-*γ*) activates STAT1 and STAT2 with consequent transcription of interferon-stimulated genes (ISGs) [50]. Among them, guanylate binding protein-1 (GBP-1) prevents epithelial apoptosis and regulates TJ integrity [51]. Other interleukins (ILs), such as IL-1*β*, -6, -22 and -23, activate STAT3 signalling in ILCs, resulting in the production of mucus and antimicrobial peptides [52]. Following triggering by commensal bacteria and/or metabolites produced by the gut microbiota, mononuclear phagocytes (MNPs; mostly monocytes, macrophages and dendritic cells) react to promote oral tolerance, the mechanism of local and systemic immune system unresponsiveness to orally-introduced antigens [53]. A well-known mechanism of tolerance is determined by MNPs migration from the lamina propria, where they reside, into MLNs. Here, these cells produce IL-10 and TGF-*β* to promote differentiation of T regulatory (T_reg_) cells, such as CD4+, Tr1+ and Foxp3+, and consequent inhibition of T effector (T_eff_) cells, thus maintaining homoeostasis [18, 54]. Higher levels of IL-10 and TGF-*β*, as well as dendritic-derived B-cell activating factor (BAFF) and proliferation-inducing ligand (APRIL), also promote B regulatory (B_reg_) cells differentiation into IgA-producing plasma cells [55]. The consequent release of anti-inflammatory mediators, such as IL-10, -35 and TGF-*β*, supports immune suppression and grants intestinal homoeostasis [18, 56, 57].

On the other hand, transient epithelial barrier damage is associated with the translocation of commensal and pathogenic microbes, resulting in abnormal infiltration of immune cells and increased cytokine release [58–60]. Pathogen-associated molecular patterns (PAMPs) found on the surface of microorganisms are recognised by resident innate immune cells through surface pattern recognition receptors (PRRs), including toll-like receptors (TLRs) and NOD-like receptors (NLRs) [61]. As a result, chemokines and cytokines are released, promoting neutrophil recruitment and phagocytosis of invading pathogens [36, 62]. Apoptotic and necrotic epithelial cells release debris, establishing the damaged-associated molecular patterns (DAMPs), which constitute an additional layer of pro-inflammatory signalling, known as “sterile” inflammation [63] (Fig. 4, middle panel). Additionally, tumour necrosis factor-alpha (TNF-*α*)-mediated activation of NF-kB and the MAPK pathways stimulate cytokines production and adaptive T cells maturation into T helper (Th)-1 (Th1), Th2 and Th17 [52, 59, 64]. The same lineages can also differentiate through IL-4, -6, -10, -12 and TGF-*β* released by dendritic cells [55]. The mechanisms that permit the resolution of such transient inflammation episodes are still unclear. Animal models of transient colitis have shown that trans-differentiation of Th17 into T_reg_ lineages underpins the resolution of gut inflammation [65]. However, these findings have not yet been validated in humans, where the established resolutive pathways likely rely on the interplay between neutrophil apoptosis, anti-inflammatory cytokines produced by T_reg_ cells (e.g. IL-10 and TGF-*β*) and macrophage efferocytosis [66, 67].

Chronic inflammation, as occurs in IBD, has a complex aetiology and engages a wide repertoire of immune responses, including Th1, Th2 and Th17 [52]. Unsurprisingly, a profound alteration in the intestinal cytokine repertoire is key to IBD establishment. In case of persistent damage, prolonged activation of neutrophils and macrophages leads to oxidative damage and the release of inflammatory mediators. The higher release of chemokines together with the increased expression of chemokine receptors (e.g. CCR7) promotes chemokine signalling, resulting in the migration and retention of dendritic cells into inflamed regions [68] (Fig. 4, right panel). Upregulation of TNF-*α*, IL-1*β*, -6, -18, -23 and IFN-*γ* in MNPs, mesenchymal and epithelial cells is sustained by NF-kB, JAK/STAT, c-Jun N-terminal kinase (JNK) and p38 kinase signalling pathways [69]. In addition, the JNK pathway and the Fas/FasL complex contribute to increased apoptotic events, perniciously prolonging the damage to the intestinal mucosa [69]. Several cytokines, namely IL-12, -18, -21 and -27, were upregulated in tissue specimens from UC and CD patients, independent of inflammation, as non-inflamed tissue from those patients showed a similar increase in cytokine expression compared to healthy controls [70]. Moreover, the cytokine signatures have potential as biomarkers to differentiate the two types of IBD as CD primarily expresses Th1- and Th17-associated cytokines (IL-17, -23 and -32), whereas UC is an atypical Th2-with low IL-4 and high IL-5, -13, -15 and -33 [70]. In addition, higher expression of IL-9-producing cells, found in UC colon tissues and models of mice-induced colitis, established a novel Th9 phenotype, highlighting the need for further studies to define the complex immune network involved in IBD [71]. For a comprehensive review of the role of inflammatory mediators, including immune cells, gut microbiota, microRNA, inflammasome and DAMPs, the reader is referred to [68].

### ECM proteins and MMPs in disease

Following damage to the intestinal epithelium, ECM components contribute to regulating the inflammatory response and repairing the wounded area by sensing the damage and promoting immune cell infiltration (Table 2). Chemokines drive neutrophil transmigration into the wounded area by activating integrins, adhesion receptors on the cell surface of neutrophils [72, 73]. As outlined in the previous section, prolonged exposure to the intestinal cytokines in response to unresolved barrier damage stimulates neutrophils, causing them to undergo degranulation and release ECM degrading enzymes, such as Cathepsin G, neutrophil elastase (NE) and MMPs, especially collagenases and gelatinases [74]. The release of MMPs from neutrophils initiates ECM degradation, facilitating cell migration and releasing small ECM fragments that stimulate immune recruitment and tissue remodelling in a positive feedback loop [75]. ECM proteins, such as versican, fibronectin, HSPGs and hyaluronan, deposit individually or in complexes with fibrin, platelets, coagulation factors and microfibrils, forming a provisional matrix that recruits immune mediators and facilitates wound healing [76, 77]. The crosstalk between ECM components, inflammatory markers and TGF-*β* also stimulates the differentiation of MCs [78, 79]. Fibroblasts, defined as vimentin-positive and *α*-smooth muscle actin (*α*-SMA) negative cells, differentiate into myofibroblasts, *α*-SMA, smooth muscle myosin (SMM) and vimentin-positive, but desmin-negative cells. Upon differentiation, myofibroblasts acquire contractile and migratory properties [80]. Moreover, they secrete and activate MMPs to degrade the provisional ECM and release de novo ECM components [81]. The concomitant activation of crypt-associated signalling pathways mobilises neighbouring epithelial cells to temporarily restore the barrier, and, in the long term, allows the proliferation and differentiation of the ISCs to restore the damaged epithelium [37, 38, 82].

**Table 2:** ECM proteins and metalloproteinases mediating the biological processes leading to IBD.

Processes leading to IBD	ECM components involved	Ref
ECM fragmentation	MMP-2, MMP-8, MMP-9, MMP-12	[75, 131]
Recruitment and migration of immune cells	Laminin, HSPGs, MMP-8, MMP-9	[7, 60, 61, 131]
Activation of inflammatory cytokines and kinases	MMP-3, MMP-9, MMP-13	[75, 78, 131]
Wound healing	HSPGs, Fibronectin, Hyaluronan, MMP-2, MMP-10	[15, 65, 76]
Barrier permeability	MMP-2, MMP-7, MMP-9, MMP-12, MMP-13	[15, 75, 77, 125, 131, 132]

Several studies have confirmed that alterations of the epithelial-mesenchymal-ECM interplay underpin the increase in intestinal permeability, with a significant association with imbalanced ECM proteolytic activity [83]. Following increased MMP expression, ECM homoeostasis is impaired and can result in excessive deposition of ECM, increased fragmentation, and irregular distribution towards tissue margins [84]. Therefore, wound healing fails, leading to sustained inflammation and fibrosis [85]. In IBD, increased activity of MMPs has been observed in patients and both in in vitro and in vivo models of the disease [86]. In IBD patients, higher levels of several MMPs have been observed [87, 88]. Imbalances in MMPs have been observed with colitis-associated colorectal cancer, which affects around 2% of individuals facing IBD [89]. High levels of MMPs relate to increased cellular extravasation, angiogenesis, immune evasion, and apoptotic resistance via degradation of ECM, blood vessels, cytokines, and apoptotic factors, which result in tumour survival and metastasis (reviewed by [90]). The contribution of MMPs to disease pathophysiology has been demonstrated in animal models. For example, mice deficient in MMPs are resistant to dextran sulfate sodium (DSS) and 2,4,6-trinitrobenzene sulfonic acid (TNBS) induced colitis [87, 88, 91].

#### MMP-2 and MMP-9

The activity of MMP-2 and MMP-9 has been reported to cause higher ECM fragmentation and to reduce tissue re-epithelialization [92]. MMP-9, along with MMP-8, fragments collagen to form proline-glycine-proline (PGP) peptides, whose structural similarity to IL-8 promotes the CXCL8-CXCR1/2 inflammatory pathway, facilitating chemotaxis [7, 19, 73]. In addition, PGP peptides can also act as inducers of MMP-9 expression and further promote neutrophil migration and differentiation, as proved in DSS-colitis models [93, 94]. Enhanced expression of MMP-9 has been implicated in the formation of complexes with neutrophil gelatinase-associated lipocalin (NGAL), an ECM component released from neutrophil granules [95]. NGAL/MMP-9 complexes have been found to increase in the serum of CD and UC patients [96, 97]. It has been hypothesised that this complex protects MMP-9 from degradation, resulting in enhanced proteolytic activity [98, 99]. MMP-9 has also been associated with TIMP-3. TIMP-3-KO mice display increased MMP-9 and ADAMs *α*-secretase activity, leading to activation of the TNF-*α* converting enzyme (TACE), which augments the production of circulating TNF-*α* and prolongs the inflammatory features of IBD [29].

**Fig. 5:**
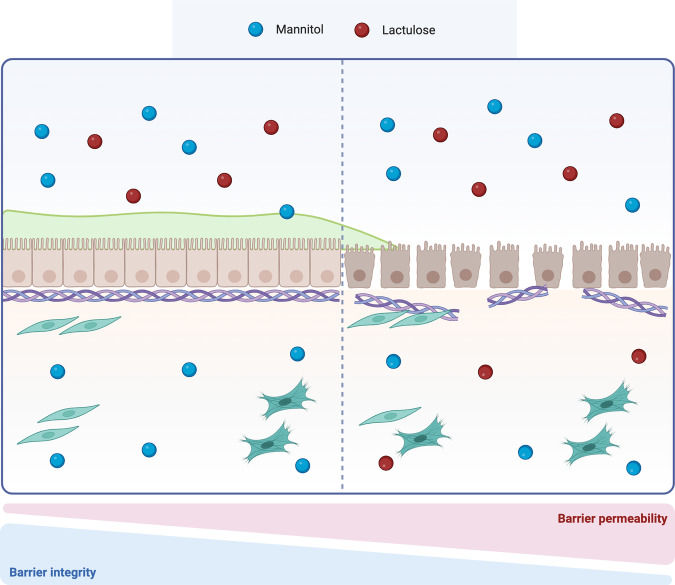
Lactulose to mannitol ratio. The lactulose to mannitol ratio (LMR) is an indicator of barrier integrity. Lactulose is a slightly absorbed disaccharide that undergoes elimination, while mannitol is a polyol highly absorbed in the intestinal mucosa. Higher levels of LMR suggest the presence of a functional and integer intestinal barrier, however, when this ratio is lower, barrier disruption is expected [136]. Created with BioRender.com.

Increased activation of MMPs is linked to epithelial barrier leakiness, as observed in UC patients, where higher levels of MMP-9 and -2 were associated with lower lactulose to mannitol ratio in urine, an indicator of higher barrier permeability [100] (Fig. 5). Recently, Al-Sadi et al. have explained a different mechanism of increased barrier permeability, where MMP-9 is implicated in the activation of myosin light chain kinase (MLCK), an enzyme responsible for the phosphorylation of myosin light chain (MLC), a regulator of perijunctional actinomyosin contractility. In their study, MMP-9 has been found to increase MLCK expression in a p38-dependent fashion [86]. The association between MMP-9, p38 kinase and MCLK is likely mediated by the pro-inflammatory transcription factor NF-kB, as silencing of the p38 kinase prevented MMP-9 from activating NF-kB p65 and increasing MLCK expression [91]. In addition, MMP-9 may affect the mucus layer surrounding the intestinal epithelium, where MUC2 is the most relevant component and acts as a marker of mucosal robustness. MMP-9-deficient mice display higher production of MUC2 at the mRNA and protein levels, which correlates with increased differentiation of intestinal cells towards the secretory lineages. On the other hand, in the goblet cell line HT-29-cl.16E, MMP-9 overexpression decreased MUC2 and altered mucins, suggesting a pivotal role for this protease in regulating goblet cells’ activity [101].

#### MMP-7

Increased MMP-7 expression has also been linked to barrier dysfunction. In particular, Xiao et al. demonstrated that increased expression of MMP-7 was inversely related to claudin-7 expression in murine models and IBD patient tissues. In this study, treatment of colonic epithelial cell lines with MMP-7 resulted in the cleavage of Claudin-7 and increased barrier permeability in vitro. Moreover, MMP-7 knockdown ameliorated inflammatory markers, including IL-6, IL-1*β*, and TNF-*α* in DSS mice, as well as *Muc2* expression. Despite unaltered *Cldn7* mRNA expression, MMP-7 KO animals displayed significantly higher levels of claudin-7, confirming that MMP-7 fragments Claudin-7 post-translationally [102].

#### MMP-12 and MMP-13

Studies focusing on the macrophage-secreted MMP-12 found that knockout mice presented reduced susceptibility to acute and chronic DSS-induced colitis. Lack of MMP-12 also led to reduced laminin fragmentation at the basement membrane level, lower occludin and claudin expression, and MLC phosphorylation by MLCK. Additionally, MMP-12 induced macrophage migration in a Caco-2 and U937 macrophages in vitro co-culture model [103].

MMP-13 has also been observed in IBD patients, where the protease was increased in the inflamed tissue compared to non-inflamed areas [104]. Recent findings showed how MMP-13, activated by TNF-*α* release, disrupts TJs and reduces MUC2 expression. This finding was supported by evidence from MMP-13 Knockout mice challenged with DSS, where neither junctional nor mucosal damage was observed [105].

#### Other intestinal proteases implicated in IBD

In addition to metalloproteinases, a wide range of intestinal proteases, such as serine- (Neutrophil elastases (NE), tryptases, cathepsin G), cysteine- (Caspases), and luminal- (bacterial-derived) proteases, contribute to increased proteolytic activity and consequent barrier leakiness [12]. In UC patients, higher NE elastolytic activity has been reported, consistent with what was observed in DSS and TNBS mice models [6, 106, 107]. Exogenous administration of elafin, an elastase inhibitor produced by epithelial cells, ameliorated disease progression by decreasing NE expression, pro-inflammatory cytokines and ZO-1 disruption, and reducing mucosal damage in mice [107]. Motta et al. have also investigated an IBD detrimental elastolytic activity linked to epithelial elastase 2A (ELA2A). In vitro studies conducted on HT-29 and Caco-2 cell lines highlighted the role of ELA2A in increasing epithelial permeability, which was found to be prevented by elafin administration [6]. Among the serine proteases, trypsin and cathepsin G have been linked to increased activation of protease-activated receptors (PARs) [108, 109]. An inverse ratio was observed between increased PAR-1 and PAR-2 and decreased ZO-1, suggesting that active degrading properties and increased paracellular permeability are mediated by these enzymes [110, 111]. Studies conducted on specimens from UC and CD patients highlighted the relevance of bacterial-derived proteases in degrading the ECM. In both UC and CD, 25% of the samples showed a significant increase in *C. perfringens*, whose MMPs drove the degradation of collagen type IV and led to increased intestinal permeability [112]. These findings suggest that a large variety of degrading enzymes are involved in controlling ECM proteolytic activity and barrier integrity and that a deeper investigation of their functions is warranted to further our understanding of IBD pathophysiology.

## Discussion

The interplay between epithelial cells, the underlying stromal compartment and the ECM forms a dynamic network pivotal to protecting, repairing, and renewing the intestinal mucosa. This sophisticated interaction prevents the infiltration of damaging pathogens, allows the passage of nutrients and other harmless substances, and maintains a core balance between immune cells and inflammatory mediators. In this context, matrix metalloproteinases appear to be a converging element of communication, key to protecting intestinal homoeostasis. The proteolytic activity of MMPs has been observed in several physiological processes regulating the genesis, repair and remodelling of blood vessels and tissues. However, under pathological conditions, dysregulated MMP expression and activity enhance tissue degradation.

In IBD, MMP-2, -7, -9, -12 and -13 have been implicated in ECM protein fragmentation, altered barrier contractility, degraded tight junctions, and compromised mucus layer, leading to higher intestinal permeability. These pathological features have been observed in both in vitro and in vivo studies, as well as in patient samples. Specific alterations in MMPs and immune factors distinguish IBD from other intestinal pathologies and can also be used to differentiate Crohn’s disease from Ulcerative Colitis (Table 1) [8]. Higher MMP-9 serum levels have been related to Crohn’s disease relapses [113], while elevated plasma levels of MMP-2, -9 and -13 have been addressed as potential biomarkers of colorectal cancer [114–116]. Research findings have shown that Crohn’s disease is characterised by Th1 and Th17 inflammation, whereas Ulcerative Colitis is characterised by an atypical Th2 response [70]. Sparano et al. showed that only MMP-11 is currently used as part of a prognostic test (OncotypeDX) for breast cancer [117]. However, in the context of IBD and colorectal cancer MMPs’ biomarker studies have not yet provided a useful tool for diagnostic or therapeutic purposes. This highlights the complexity of IBD and the need to dissect the crosstalk between MMPs, the immune environment and barrier integrity [118]. Since the enhanced activity of MMPs has been well documented in IBD patients, several attempts have been made to inhibit MMPs, but have demonstrated low efficacy [95, 119]. In the context of transient inflammation, inhibition of MMPs is mediated by TIMPs. However, in UC and CD, increased levels of MMPs can occur even in cases with concomitant higher expression of TIMPs (e.g., in fibrotic disease), suggesting that MMPs’ increased levels cannot be counteracted by TIMPs’ activity [120]. Guedez et al. have demonstrated the potential of TIMP-2 to inhibit tumour proliferation in lung cancer models. TIMP-2 deficiency favoured the recruitment of cancer myeloid-derived suppressor cells (MDSC) by promoting angiogenesis-associated tumour growth and immunosuppressive cytokines and chemokines [121]. In this context, further studies should aim at investigating changes in the ratio between MMPs and TIMPs in different clinical conditions. Pharmacological inhibitors of MMPs have been employed in numerous in vitro and in vivo studies that aimed at treating IBD [122]. Batimastat and marimastat were designed to mimic collagen, bind MMPs and avoid degradation of ECM proteins [90]. After reaching clinical trials phase I and II/III, respectively, they showed significant musculoskeletal syndrome; therefore, further investigations were ceased [123, 124]. The reasons why MMPs inhibitors have not delivered promising results include the unclear understanding of MMPs pharmacokinetics and pharmacodynamics [125]. In addition, current drug discovery studies lack proper biochemical targeting. For example, MMPs inhibitors addressing cancer metastasis have broad-spectrum proteolytic activity and act at disease stages where MMPs are poorly involved, leading to uncontrolled proteolysis and unsuccessful outcomes [90].

The effect of other proteases on barrier function has also been investigated. For example, the inhibition of neutrophil elastases and serine proteases, which target the epithelium and its underlying support structure, has demonstrated a restorative effect on barrier permeability and intestinal inflammation [107]. It would be interesting to investigate whether this protective mechanism is a result of changes in ECM remodelling.

### Future perspective

To date, impaired barrier permeability has been highlighted as the initiating factor for pathogenic infiltration into the intestinal mucosa and subsequent chronic inflammation due to exposure to PAMPs. However, the unbalance between pro- and anti-inflammatory pathways also contributes to the failed resolution of acute inflammation, and its consequent chronic inflammation [63]. Therefore, investigating anti-inflammatory immune cells, cytokines and their signalling pathways represents an alternative approach to developing treatments towards IBD. Recent studies have also targeted pathogenic bacterial-derived proteases, whose increased expression enhances ECM proteolysis, worsening IBD inflammation and pathophysiology [112]. In this context, it is natural to wonder if there is a crosstalk between MMPs and bacterial-derived proteases, and whether they might have an additive effect during IBD. Additionally, it would be interesting to investigate microbial phyla known to be beneficial, to understand whether they might reverse ECM remodelling and/or influence the increased proteolytic activity observed in IBD.

An additional confounding factor that limits research advancement is the model systems currently used in IBD research. The animal and cell-based models used for such studies do not allow a consistent and reliable recapitulation of the human disease. These current limitations outline the need for targeted studies that take a reductionist approach and allow better control of experimental variables. Achieving these outcomes offers an opportunity for future studies, especially in the context of developing novel in vitro models recapitulating the intestinal mucosa under healthy and diseased conditions. This could reduce the use of animal models in IBD research, which have physiological and ethical limitations. In this context, 3D models can represent a flexible tool to dissect the complexity of the intestinal epithelium in a controlled environment. This might be achieved by the subsequential addition of single variables (e.g., microbiome components and environmental inflammatory triggers) followed by the investigation of their individual effects. In addition, 3D models could be generated by the co-culture of cells derived from patient tissues, paving the way to precision medicine studies [126].
